# miR-377-3p-Mediated EGR1 Downregulation Promotes B[a]P-Induced Lung Tumorigenesis by Wnt/Beta-Catenin Transduction

**DOI:** 10.3389/fonc.2021.699004

**Published:** 2021-08-23

**Authors:** Xinxin Ke, Lulu He, Runan Wang, Jing Shen, Zhengyang Wang, Yifei Shen, Longjiang Fan, Jimin Shao, Hongyan Qi

**Affiliations:** ^1^Department of Pathology and Pathophysiology, and Department of Radiation Oncology of the Second Affiliated Hospital, School of Medicine, Zhejiang University, Hangzhou, China; ^2^Department of Pathology and Pathophysiology, and Department of Medical Oncology of the Second Affiliated Hospital, School of Medicine, Zhejiang University, Hangzhou, China; ^3^Department of Pulmonary and Critical Care Medicine, Sir Run Run Shaw Hospital, School of Medicine, Zhejiang University, Hangzhou, China; ^4^Institute of Crop Science and Institute of Bioinformatics, Zhejiang University, Hangzhou, China; ^5^Key Laboratory of Disease Proteomics of Zhejiang Province, Key Laboratory of Cancer Prevention and Intervention of China National Ministry of Education, and Research Center for Air Pollution and Health, School of Medicine, Zhejiang University, Hangzhou, China

**Keywords:** early growth response protein 1, miR-377-3p, malignant transformation, lung tumorigenesis, Wnt/β-catenin pathway

## Abstract

Polycyclic aromatic hydrocarbons (PAHs), particularly benzo[a]pyrene (B[a]P), found in cigarette smoke and air pollution, is an important carcinogen. Nevertheless, early molecular events and related regulatory effects of B[a]P-mediated cell transformation and tumor initiation remain unclear. This study found that EGR1 was significantly downregulated during human bronchial epithelial cell transformation and mice lung carcinogenesis upon exposure to B[a]P and its active form BPDE, respectively. In contrast, overexpression of EGR1 inhibited the BPDE-induced cell malignant transformation. Moreover, miR-377-3p was strongly enhanced by BPDE/B[a]P exposure and crucial for the inhibition of EGR1 expression by targeting the 3’UTR of EGR1. MiR-377-3p antagomir reversed the effect of EGR1 downregulation in cell malignant transformation and tumor initiation models. Furthermore, the B[a]P-induced molecular changes were evaluated by IHC in clinical lung cancer tissues and examined with a clinic database. Mechanistically, EGR1 inhibition was also involved in the regulation of Wnt/β-catenin transduction, promoting lung tumorigenesis following B[a]P/BPDE exposure. Taken together, the results demonstrated that bBenzo[a]pyrene exposure might induce lung tumorigenesis through miR-377-3p-mediated reduction of EGR1 expression, suggesting an important role of EGR1 in PAHs-induced lung carcinogenesis.

## Introduction

Lung cancer has the highest morbidity and mortality worldwide. Late diagnosis and poor prognosis are the main causes of cancer-related death (1, 2), and smoking is a common risk factor. Yet, over the years, the increased non-smoking-related risk associated with ambient air pollution has been frequently reported (3). Polycyclic aromatic hydrocarbons (PAHs) are widespread environmental pollutants that have been associated with carcinogenicity (in gas or particle phase) (4). The most widely studied PAH is Benzo[a]pyrene (B[a]P), which is frequently chosen as a substitute for evaluating the carcinogenic PAHs (5).

B[a]P is a human group 1 carcinogen capable of initiating and promoting lung tumorigenesis (6). BPDE is the main biologically active metabolite of B[a]P that can form DNA adducts of guanine N2, thus exerting its carcinogenic effect (7). In cell-based models, B[a]P or its metabolite BPDE induce cell malignant transformation, while in mice models, it can reduce lung tumors. Recent studies have shown that B[a]P-induced tumorigenesis involves DNA methylation, oxidative stress, cell cycle, inflammation, apoptosis, and other biological processes (7–9). Yet, the exact molecular mechanism behind this remains unclear.

Transient activation and regulation of immediate-early genes are considered primary cellular responses to an external signal in cancer development (10). Early growth response 1 (EGR1) is an immediate-early gene that can be directly activated by growth factors, hypoxia, ischemia, tissue injury, and apoptotic signals in different cells (11). Different roles of EGR1 have been observed in different tumors. EGFR1 can have double-edged effects in tumor development. For example, EGR1 has an oncogenic function in prostate cancer by promoting cell proliferation and survival, but it can also act as a tumor suppressor in various cancers such as glioma, lung, and bladder cancer by directly upregulating PTEN, P53, and fibronectin (12–16).

MicroRNAs (miRNAs), an endogenous short non-coding RNA, have important functions in many developmental systems (17). miRNAs regulate gene expression in multicellular organisms by affecting both the stability and translation of mRNAs. They can target the 3’-UTR of mRNA transcripts *via* complementary sequences and repress the gene expression by post-transcriptional level (18). Their deregulation has been closely related to cancer initiation and progression (19).

miR-377-3p is a novel tumor regulatory miRNA whose biological functions are wildly unknown. MiR-377-3p has been shown to possess tumor-inhibiting effects in clear cell renal cell carcinoma and hepatocellular carcinoma (20, 21). Contrary, previous studies have shown that miR-377 promotes the proliferation and EMT process in colon cancer, while the low level of miR-377 was associated with a good prognosis of periampullary adenocarcinoma (22, 23). Moreover, recent studies demonstrated that miRNAs are also involved in B[a]P-induced carcinogenicity (24, 25). However, the potential contribution of miRNAs in environmental carcinogens-induced lung tumorigenesis is still not clear.

In the present study, we found that EGR1 expression was strongly reduced in the malignant transformation of human lung bronchial epithelial cells and lung tumorigenicity following B[a]P and its active metabolite BPDE exposure. Moreover, miR-377-3p mediated EGR1 downregulation facilitates cell malignant transformation and tumor formation by regulating the Wnt/β-catenin pathway, suggesting an important role of the miR-377-3p/EGR1 axis in the malignant transformation of lung tumorigenesis induced by environmental carcinogen.

## Materials and Methods

### Patient Samples

A total of 114 non-small-cell lung cancer (NSCLC) clinical samples of the Second Affiliated Hospital of Zhejiang University were used in this study. The study was approved by the ethics committee of the hospital. The clinical characteristics of these samples are shown in **Table 1**. The cancer tissues were formalin-fixed and paraffin-embedded for immunohistochemistry (IHC).

**Table 1:** Association of immunohistochemical staining for EGR1 with the tumor clinic pathological characteristic.

Clinicopathological features	Case N. (%)	Egr1 expression		*p*-value
		High	Low	
Gender				
Male	63 (55)	27	36	
Female	51 (45)	20	31	0.296
Age				
Mean (Range)	62 (37-85)			
<60	65 (57)	26	39	
>60	49 (43)	21	28	0.476
Tumor size^a^				
<2.5cm	46 (51)	21	25	
>2.5cm	45 (49)	19	26	0.565
Depth of invasion				
T1	46 (40)	38	16	
T2	53 (46)	28	25	
T3	11 (10)	1	10	
T4	4 (4)	2	2	0.013*
Lymph node metastasis				
N0	50 (44)	31	19	
N1	29 (25)	7	22	
N2	35 (31)	9	26	0.000**
Distant metastasis				
M0	107 (94)	44	63	
M1	7 (6)	3	4	0.750
TNM stage				
I	44 (39)	29	15	
II	29 (25)	8	21	
2III	36 (32)	8	28	
IV	5 (4)	2	3	0.000**
Histological grade^b^				
High	31 (28)	19	12	
Moderate	63 (58)	25	38	
Poor	15 (14)	3	12	0.003*

^a^23 cases without tumor size.

^b^5 cases without tumor histological grade.

*P < 0.05 and **P < 0.01.

### Cells and Reagents

Human bronchial normal epithelium cell BEAS-2B (Cell Bank of the Chinese Academy of Science, Xiangya, China) and 293T cells (ATCC, Manassas, VA, USA) were cultured in DMEM (Gibco, Grand Island, NY, USA.) supplemented with 10% FBS (Gibco), streptomycin (100 g/mL), and penicillin (100 U/mL) in a humidified atmosphere containing 5%CO_2_/95% air at 37°C. The authenticity of the cell lines used in this study has been verified by STR profiling.

BPDE was purchased from the National Cancer Institute Chemical Carcinogen Reference Standard Repository (Kansas City, MO, USA), dissolved in DMSO, and stocked in -80°C.

### Cell Transformation Assays

Cells were exposed to 0.2 µM or 0.5 µM BPDE for 2 hours in a serum-free medium. Then, the treated medium was removed, and cells were recovered in a fresh medium at 37°C. BPDE exposure was repeated once a week for 12 weeks. After 12 weeks of treatment, the malignant phenotype was analyzed and DMSO was used as solvent control.

### QRT-PCR

Total RNA was extracted from cell lines or tumor and normal tissue samples with TRIzol reagent (Invitrogen, Carlsbad, CA, USA). For gene expression, RNA was reverse transcribed using a Prime-Script RT reagent Kit (TaKaRa). QRT-PCR was carried out with an SYBR Premix Ex Taq (TaKaRa). For miRNA expression, RNA was reverse transcribed using an SYBR^®^ Premix Ex Taq II (TliRnaseH Plus) (TaKaRa, Dalian, China). QRT-PCR was performed using a Mir-X miRNAFirst-Strand Synthesis kit (Clontech, Madison, WI, USA). Experiments were performed in triplicate, and the values were normalized to GAPDH or RNU6B using the 2(−ΔΔCt) method for gene and miRNA expression analysis, respectively.

### Immunoblot Analysis

Cell lysates (50 mg) were separated on a 10% SDS-PAGE gel and then transferred onto a nitrocellulose membrane (Whatman, Maidstone, UK). The membrane was blocked with 5% skim milk solution for 2 hours and incubated overnight at 4°C with the following diluted primary antibody: rabbit monoclonal anti-human EGR1 (ab194357, Abcam, Hanghzou, China), and the mouse monoclonal anti-human GADPH (sc-47724) and anti-human β-catenin (sc-7963) both purchased from Santa Cruz Biotechnology (Santa Cruz, CA, USA). Then, the membrane was incubated in IRDye^®^ 800CW- or IRDye 680-conjugated secondary antibody (LI-COR Biosciences, Lincoln, NE, USA) and detected by an Odyssey^®^ infrared imaging system.

### Animal Models

A/J mice (4 weeks) and Balb/c nude mice (4 weeks) were obtained from Model Animal Research Center, Nanjing, China and SLAC Laboratory Animal, Shanghai, China. All the animals were housed in an environment with a temperature of 22 ± 1°C, relative humidity of 50 ± 1%, and a light/dark cycle of 12/12 h. All animal studies (including the mice euthanasia procedure) were done in compliance with Zhejiang University institutional animal care regulations and guidelines, and conducted according to the AAALAC and the IACUC guidelines.

A/J mice (4 weeks) were randomly divided into two groups (12 mice/group). B[a]P group was intraperitoneally injected with B[a]P (25 mg/kg, in tricaprylin solvent) (Sigma), and the control group was intraperitoneally injected with the tricaprylin solvent. B[a]P treatment was given on a weekly basis for 8 weeks. The control group was treated as the same. After 4 months of restoration following the treatment period, mice were sacrificed, and the lung tissues were obtained and histologically examined. The tricaprylin solvent-treated group was used as a control group.

Balb/c nude mice (4 weeks) were subcutaneously injected with 5 × 10^6^ transformed cells in 100 µl volume mixed with Matrigel (1:1). Three days after injection, miR-377-3p antagomir (5 nmol/mouse) or scramble control was performed by intratumor injection twice a week. The long diameter (a) and short diameter (b) of the tumors were measured; after which, the volume (V) was calculated using the formula V = 1/2 × a × b2. Mice were sacrificed, and the tumor tissues were obtained and weighed.

### Soft Agar Assay

The cells (1,000 cells/well) were suspended in a culture medium containing 0.4% agarose (Sigma, St Louis, MO, USA) and seeded onto a base layer of 0.7% agar bed in 12-well plates. After 2 weeks, colonies were stained with crystal violet and photographed. Colonies ≥ 0.05 mm in diameter were counted.

### Scratch Test

Cells (1 x 10^5^ cells/ml) were plated in 6-well plates. The monolayer was scratched by a 10 ml sterile pipette tip. The cells were gently rinsed twice with PBS to remove floating cells and incubated in 2 ml of serum free medium in 37°C, 5% CO2 air environment. Images of the scratches were taken by using an inverted microscope at 0, 24, and 48 hours of incubation. ImageJ software was used to analyze the percentage of wound closure.

### Transwell Assay

We performed a cell migration assay with an 8 µm-pore in 24-well transwell plates (Costar, Cambridge, MA, USA). Briefly, 400 ml of complete DMEM medium was added under the chambers, whereas cells (2 × 10^4^) were added above the chambers in a serum-free medium. After 48 hours of incubation at 37°C, the migrated cells were fixed with 4% paraformaldehyde and stained with 0.5% crystal violet. Then, the filter membrane was examined and photographed under a microscope.

### Immunohistochemistry

The IHC was performed using an Envision Detection System (DAKO, Carpinteria, CA) according to the instructions of the manufacturer. Rabbit monoclonal anti-mouse Ki67 (ab194357) was purchased from Abcam; rabbit polyclonal anti-mouse EGR1 (sc-110) was purchased from Santa Cruz Biotechnology. The IHC staining results were assessed and confirmed by two independent investigators blinded to the clinical data.

### Cell Transfection

For lentiviral-mediated transfection, 293T cells were co-transfected with the lentiviral and packaging vectors. After 72 h, the supernatant was collected. Supernatants were then collected and centrifuged at 1,000 × g for 15 min at 4°C to pellet debris. Before performing the infection, the lentiviruses were recovered and re-suspended in a fresh medium with 6 g/ml of polybrene. Stable cells with EGR1 knockdown or EGR1 overexpression were selected following transduction with 0.5 mg/ml of puromycin for 2 weeks. Transfection efficiency of EGR1 knockdown or EGR1 overexpression was examined by Western blot.

For miR-377-3p mimic, inhibitor, antagomir, miRNA control (GenePharma, Shanghai, China) transfection, cells were transfected using Lipofectamine^®^ RNAiMAX (Invitrogen) following the instructions of the manufacturer. After 72 h of transfection, the cells were collected for further experiments.

### Dual-Luciferase Reporter Assay

The full-length and mutated miR-377-3p recognition elements of 3’UTR-EGR1 were synthesized and constructed into a pGL3-Basic vector (Promega, Madison, WI, USA). After seeding the cells for 24 h, the mimic or inhibitor of miR-377-3p (GenePharma) was co-transfected with either pGL3-EGR1-3’UTR wild-type or mutant into BEAS-2B and 293T cells. Dual-Luciferase Reporter Assay System was used for testing the relative luciferase activity (Promega).

### Immunofluorescence

The BPDE-transformed cells were plated in culture. After overexpression of EGR1, the cells were fixed for 15 min in 4% formaldehyde solution. Then, the cells were washed with PBS and treated with 0.1% Triton X-100 in PBS for 10 min. After permeabilizing the cells, we blocked the cells for 1 h in an antibody blocking buffer (10% normal goat serum, 1% BSA in PBS). Then, the cells were washed with PBS and incubated with anti-human β-catenin primary antibody. The presented IF staining pictures are the overlaid images of β-catenin staining in green fluorescence with nuclear 4’6-diamidino-2-phenylindole (DAPI) staining in blue fluorescence. The IF staining images were taken and overlaid using the Nikon NIS-Elements software.

### Statistical Analysis

The two-tailed Student’s t-test and one-way analysis of variance were used for statistical data analysis. The data was expressed of three separate experiments, as mean ± standard deviation (SD). *P* ≤ 0.05 was considered to be statistically significant.

## Results

### BPDE/B[a]P Downregulates the Expression of EGR1 *In Vitro* and *In Vivo*

**Figure 1:**
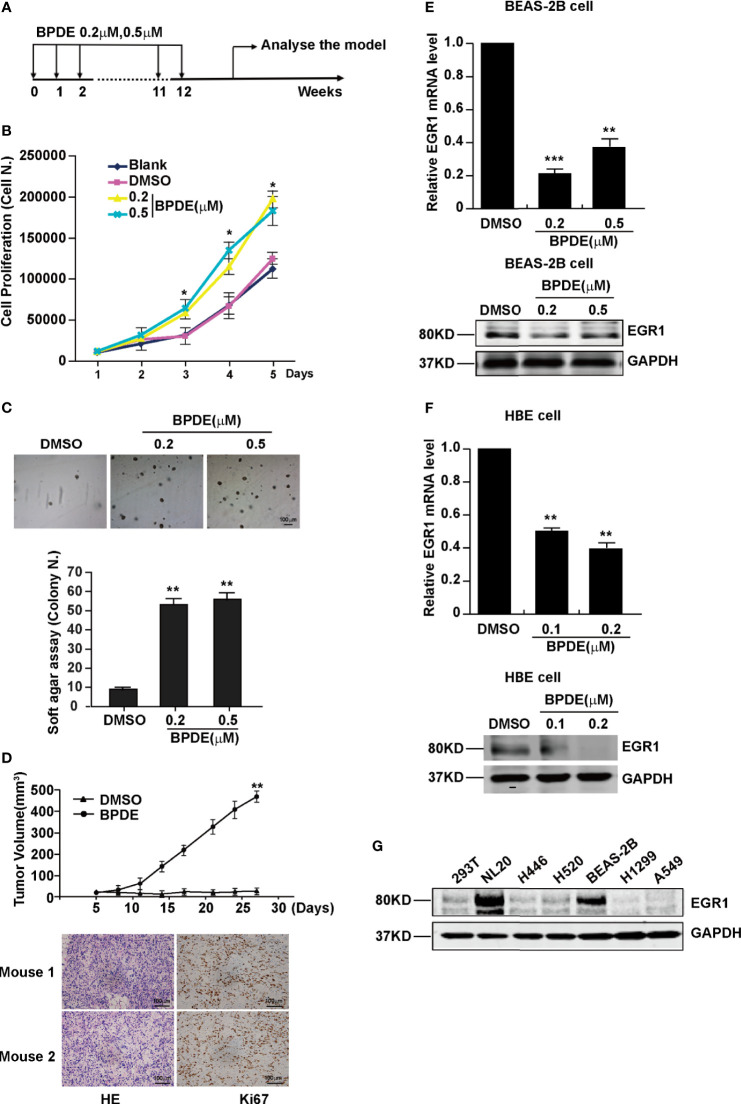
EGR1 was downregulated in BPDE-induced malignant transformation of normal human lung epithelial cells. **(A)** Schematic map of BPDE-induced malignant transformation model. **(B)** Cell proliferation ability of BPDE-treated and control cells. **(C)** Cell anchorage-independent growth in soft agar. Top, representative images; bottom, quantitative results of cell colony per field. **(D)** Top, Tumor growth curve of the transformed cells induced by BPDE and control cells injected subcutaneously in nude mice (n = 12 tumors per group); bottom, representative photos of the HE staining and Ki67 immunohistochemical staining of the tumor mass. **(E, F)** EGR1 expression analyzed by qRT-PCR and Western Blot in transformed BEAS-2B and HBE cells. **(G)** Egr-1 expression in different lung epithelial and lung cancer cells. Normal bronchial epithelial cell: NL-20, BEAS-2B; lung cancer cell: H446, H520, H1299, A549. The analyses were repeated three times, and the results were expressed as mean ± SD. *P < 0.05, **P < 0.01 and ***P < 0.001.

B[a]P and its ultimate carcinogenic metabolite, BPDE, are the strong lung carcinogens found in tobacco smoke and air pollution (26). However, the molecular mechanisms underlying PAH-induced lung tumorigenesis, particularly in the early stage, remain unclear. To indicate the critical genes involved in this process, human lung epithelial cells and A/J mice were exposed to BPDE/B[a]P, respectively. Malignant transformation of BEAS-2B cells was identified upon 12 weeks of BPDE exposure (**Figures 1** and **S1**). **Figure 1A** shows a schematic map of the strategy used to generate the BPDE-induced malignant transformation of BEAS-2B cells. Cell proliferation assay and soft agar assay revealed that BPDE treatment enhanced the reproductive capacity of cells and the anchorage-independent growth capability, respectively (**Figures 1B, C** **)**. We also observed that the cell migration was enhanced upon BPDE treatment (**Figures S1A, B**). Xenograft assay further confirmed the malignant phenotype of BPDE-induced BEAS-2B cells (**Figure 1D**). In addition, we also confirmed the above tumorigenic effects with the BPDE-induced HBE malignant transformation cell model by malignant phenotype analysis (data not shown).

To investigate the genes implicated in the BPDE-induced malignant transformation process, we performed RNA-sequencing analysis. Our results showed that EGR1 was the most obviously downregulated gene in the transformed cells (**Figure S1C**). The downregulation of EGR1 expression was confirmed in both BEAS-2B and HBE BPDE-induced cell transformed models (**Figures 1E, F** **)**. Moreover, the EGR1 protein content was also reduced in different lung cancer cells contrasted with normal cells (**Figure 1G**).

**Figure 2:**
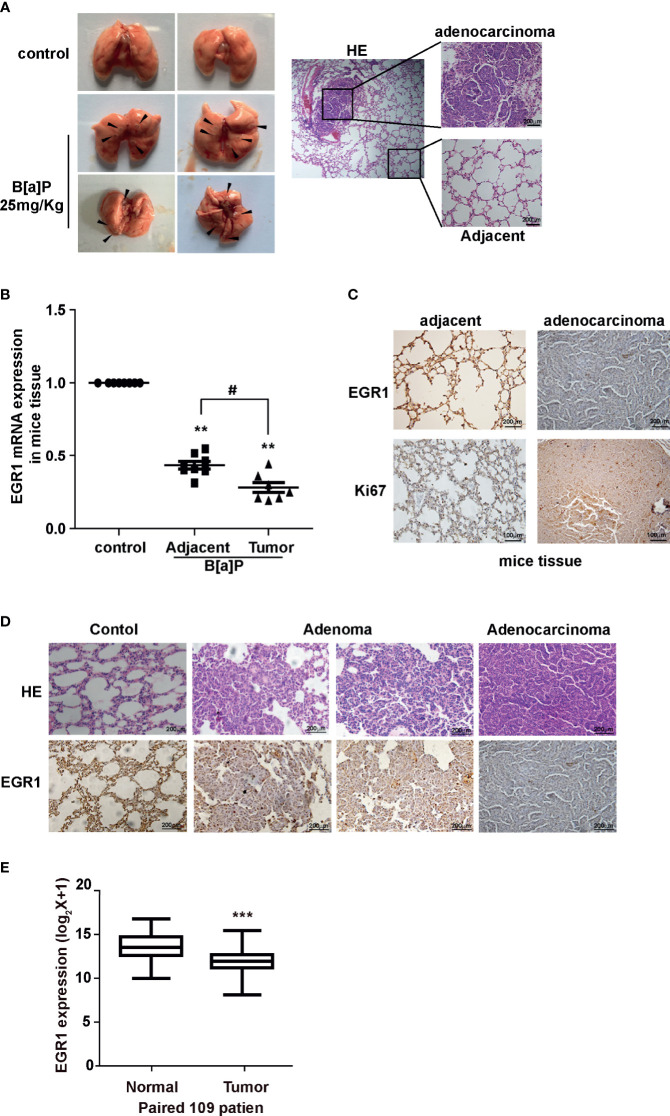
B[a]P downregulated EGR1 expression *in vivo*. **(A)** B[a]P-induced A/J mice lung tumorigenesis. Left, representative images of primary lung tumor in mice with or without B[a]P treatment (25mg/kg); right, representative images of HE staining. **(B)** EGR1 mRNA expression in B[a]P-induced murine lung tumor and adjacent normal tissues, vehicle lung tissues. **(C, D)** Representative images of EGR1, Ki67 immuno-histochemical staining of B[a]P-treated murine lung tumor tissues and adjacent tissues, vehicle lung tissues. **(E)** Analysis of the TCGA database of EGR1 mRNA expression in paired lung cancer and normal tissues. The analyses were repeated three times, and the results were expressed as mean ± SD. ^#^ *P* < 0.05, **P < 0.01, and ***P < 0.001.

To further evaluate the effect of B[a]P on EGR1 expression *in vivo*, we established a B[a]P-treated A/J mice model (**Figure S2A**). Most mice treated with B[a]P developed primary lung tumors within 6 months; this was observed by PET-CT detection and histopathological analysis (**Figures 2A** and **S2B, C**). Our results also showed that EGR1 mRNA expression and protein level were decreased in the lung tumor tissues compared to the adjacent normal tissues (**Figures 2B, C** **)**. Ki67 was extensively assessed and reported as a predictive proliferative marker of cancer cells. Moreover, the downregulation of EGR1 was not only observed in adenocarcinoma but also B[a]P-treated mice adenoma (**Figure 2D**), indicating that EGR1 reduction could be the early event in B[a]P-induced tumorigenesis.

To further determine whether EGR1 downregulation was involved in human lung carcinoma development, we expanded our study by investigating the expression of EGR1 in clinical cancer tissues. In eight pairs of fresh cancer and adjacent normal tissues from clinical NSCLC patients, we found the reduction of EGR1 expression in cancer tissues (**Figure S3A**). TCGA (The Cancer Genome Atlas) database and the other two datasets supported in Lung Cancer Explorer confirmed that EGR1 was downregulated in NSCLC patient tissues compared to normal tissues (**Figures 2E** and **S3B, C**). Collectively, the results indicated that the inhibition of EGR1 was involved in cell malignant transformation and mice lung tumorigenesis induced by BPDE/B[a]P exposure. The EGR1 reduction was also observed in clinical cancer tissues. The above data suggested that EGR1 could have a tumor-suppressive role in the lung cancer process.

### EGR1 Reduction Mediates BPDE-Induced Malignant Transformation

**Figure 3:**
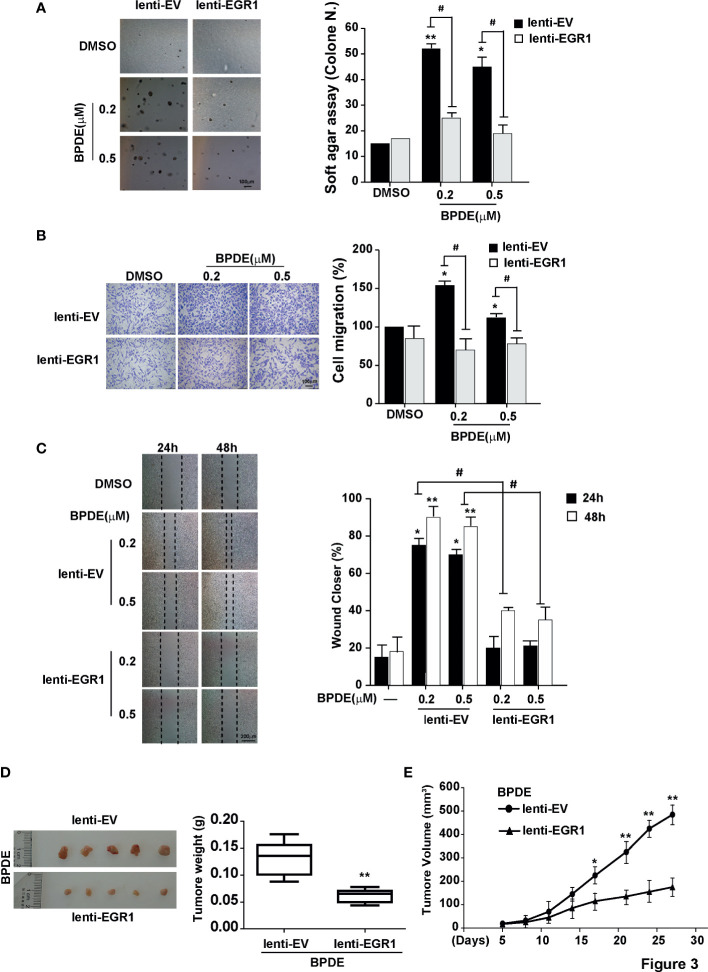
EGR1 downregulation is crucial for BPDE-induced cell malignant transformation. **(A)** Soft-agar colony formation assays of BPDE-induced transformed cells infected with EGR1 overexpression lentivirus. Left, representative images; right, quantitative results of cell colony per field. **(B, C)** Left, representative images of transwell migration and wound healing assays; right, relative numbers of migration cell and percentage wound closure after treatment. **(D)** Representative images of xenograft tumors in nude mice at 4 weeks after inoculation (Left) and the tumor weight of BPDE-induced transformed cells infected with EGR1 overexpression. **(E)** Tumor growth curve of xenograft assays (n = 10 tumors per group). The analyses were repeated three times, and the results were expressed as mean ± SD. ^#^,*P < 0.05 and **P < 0.01.

To investigate the potential role of EGR1 downregulation in lung tumorigenic effects upon BPDE exposure, we established stable EGR1 overexpression models in BPDE-induced transformed cells with lenti-EGR1 lentivirus (**Figure S4A**). The ectopic expression of EGR1 led to a reduced malignancy in BPDE-induced transformed cells (**Figure 3**). Moreover, EGR1 overexpression reduced the cell migration ability **(** **Figures 3A–C**) and xenograft tumor growth (**Figures 3D, E**). The suppressive effect of EGR1 on cell malignant phenotypes was further confirmed by EGR1 knockdown. EGR1 shRNAs introduction through lentiviral vectors resulted in an increased malignancy of BEAS-2B cells (**Figures S4C–E**). Moreover, the rescue of EGR1 also reversed the effect of EGR1-knockdown in promoting cell transformation (**Figures S4F, G**). The knockdown efficiency of EGR1 was supported in **Figure S4B**. Our results suggested that EGR1 downregulation was critical for promoting BPDE-induced cell malignant transformation.

### mir-377-3p Targets EGR1 and Induces its Inhibition Following BPDE Exposure

**Figure 4:**
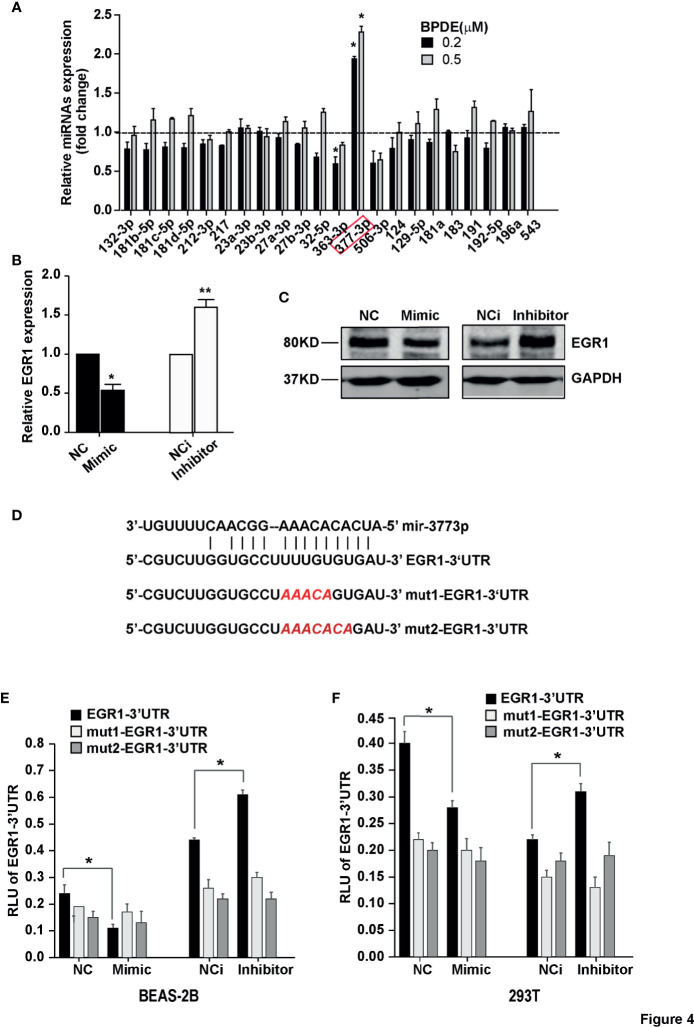
MiR-377-3p activation mediated EGR1 inhibition following BPDE exposure. **(A)** Expression profile of predicted miRNAs in BPDE-treated BEAS-2B cells by qRT-PCR. **(B, C)** EGR1 expression after transfection of miR-377-3p mimic and inhibitor in BEAS-2B cells was analyzed by QRT-PCR and Western blot. **(D)** The putative target site of miR-377-3p in the 3’UTR of EGR1 (upper panel); red letters indicate the mutant luciferase reporter gene sequence (lower panel). **(E, F)** EGR1 3’UTR luciferase reporter assays in BEAS-2B cells and 293T cells. The analyses were repeated three times, and the results were expressed as mean ± SD. *P < 0.05 and **P < 0.01.

Notably, we found that the expression of EGR1 was inhibited during the BPDE-induced cell malignant transformation (data not shown). To investigate the molecular mechanism underlying EGR1 reduction upon BPDE treatment, we first evaluated EGR1 promoter DNA methylation level by bisulfite sequencing PCR. The DNA methylation level of the EGR1 promoter sequence did not change after BPDE exposure (**Figure S3D**). Over the past decade, it has been widely reported that miRNAs regulate gene expression by recognizing the 3’UTR sequence. Using the microRNA database and target prediction tools (miRanda, PicTar, and TargetScan), we predicted the potential microRNAs that could target EGR1 and regulate its mRNA transcription. qRT-PCR revealed that miR-377-3p levels were markedly increased in BPDE-treated cells (**Figure 4A**). Transient transfection with mimics and inhibitor of miR-377-3p showed that miR-377-3p regulates EGR1 expression (**Figures 4B, C** **)**.

To further identify the effect of miR-377-3p on EGR1 expression regulation, we constructed the luciferase reporter containing wild-type regulatory sequence with or without EGR1 binding site mutation (**Figure 4D**). The result showed that miR-377-3p mimic reduced the reporter activity of the full-length EGR1 3’UTR-containing luciferase construct, and the inhibitor of miR-377-3p augmented the reporter activity in BEAS-2B and 293T cells. The effect of miR-377-3p on the reporter activity was abrogated with the mutant-type EGR1 3’UTR-containing luciferase construct (**Figures 4E, F** **)**. These results indicated that miR-377-3p mediated the downregulation of EGR1 in BPDE-induced malignant transformed cells by directly targeting its 3’UTR sequence.

### mir-377-3p Antagomir Rescued the Effect of EGR1 Downregulation in Cell Malignant Transformation and Lung Carcinogenesis

**Figure 5:**
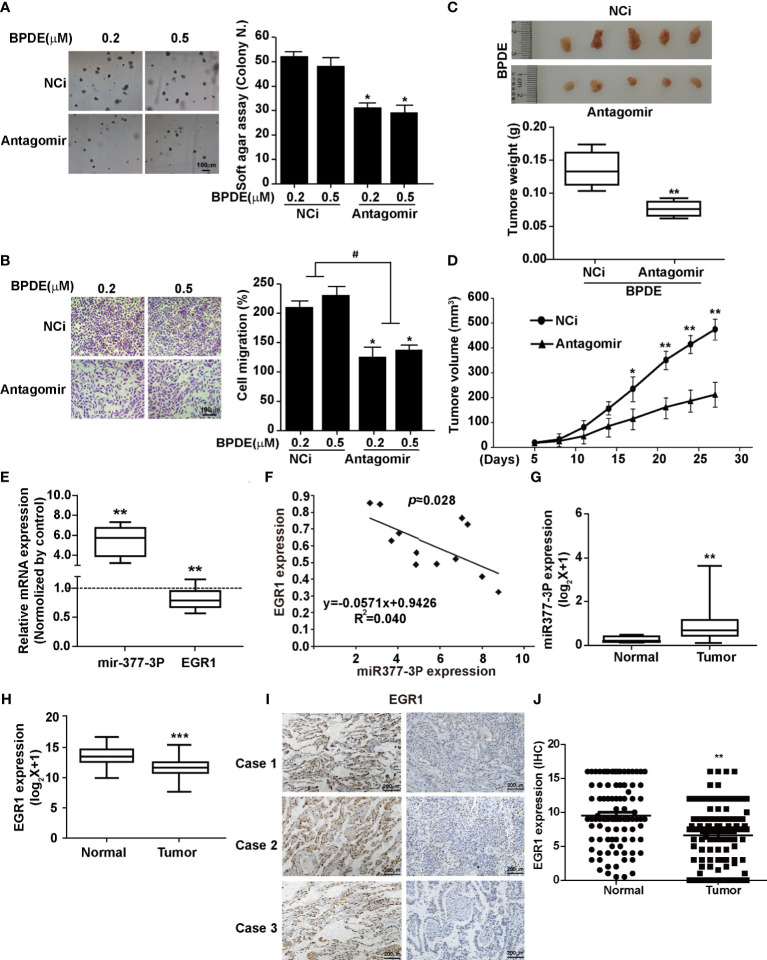
MiR-377-3p antagomir rescued the effect of EGR1 downregulation in cell malignant transformation and lung carcinogenesis. **(A)** Soft-agar colony formation assays. Left, representative images; right, quantitative results of cell colony per field. **(B)** Transwell migration assays. Left, representative images; right, quantitative results of migratory cells per field. **(C)** Representative xenograft tumor images at 4 weeks after inoculation (top) and tumor weight (bottom) of BPDE-induced transformed cells treated with miRNA-377-3p antagomir or control. **(D)** The tumor growth curve of xenograft. **(E)** MiR-377-3p expression and EGR1 mRNA expression in B[a]P-induced murine lung cancer tissues by qRT-PCR. The analyses were repeated three times, and the results were expressed as mean ± SD. **(F)** The correlation analysis of EGR1 and miR-377-3p mRNA expression in mice carcinogenesis model. Y-axis showed EGR1 mRNA expression; X-axis showed miR-377-3p mRNA expression. **(G, H)** TCGA database analysis of miR-377-3p expression and EGR1 mRNA expression in human lung adenocarcinoma tissues. **(I)** Representative image of IHC staining of EGR1 in human lung cancer tissues. **(J)** Quantification of EGR1 staining in the adjacent normal lung epithelial cells and lung cancer cells in 114 paired patient tissues. ^#^,*P < 0.05, **P < 0.01, and ***P < 0.001.

To detect whether the inhibition of miR-377-3p allows for the re-expression of EGR1 and reduces the malignancy of BPDE-induced transformed cells, we transfected the cells with miR-377-3p antagomir. Our results revealed that the antagomir of miR377-3p reduced the malignancy phenotypes of the transformed cells induced by BPDE exposure (**Figures 5A–D**). Moreover, miR-377-3p upregulation was identified in mice lung tumor tissues induced by B[a]P, concomitantly with EGR1 downregulation (**Figure 5E**). Furthermore, we observed a negative relevance between EGR1 and miR-377-3p in mice lung tumor tissues by the correlation analysis (**Figure 5F**). Consistent with our findings, the TCGA database analysis revealed the increase of miR-377-3p and the decrease of EGR1 in human lung adenocarcinoma tissues (**Figures 5G, H** **)**. Besides, the expression of EGR1 and miR-377-3p in fresh lung cancer tissues also showed a negative relevance (**Figure S5A**).

To confirm the tumor repressive effect of EGR1 in NSCLC, we performed IHC staining to evaluate the clinical relevance of EGR1 expression. Our results showed a high EGR1 immunoreactivity in the nuclei of adjacent normal cells compared with cancer cells. In 114 paired cases, EGR1 was significantly inhibited in tumor tissues (**Figures 5I, J** and **S5B**). EGR1 expression was negatively associated with tumor invasion, lymph node status, histological grade, and TNM stage (**Table 1**). Our results suggested that EGR1 functions as an onco-suppressor, and the inhibition of EGR1 was associated with tumor aggressiveness in lung cancer. Taken together, our results suggest that the upregulation of miR-377-3p inhibits EGR1 transcription, which is implicated in BPDE/B[a]P-induced cell malignant transformation and lung tumorigenesis.

### EGR1 Inhibition is Involved in the Regulation of Wnt/β-Catenin Transduction in PAHs-Induced Tumorigenesis

**Figure 6:**
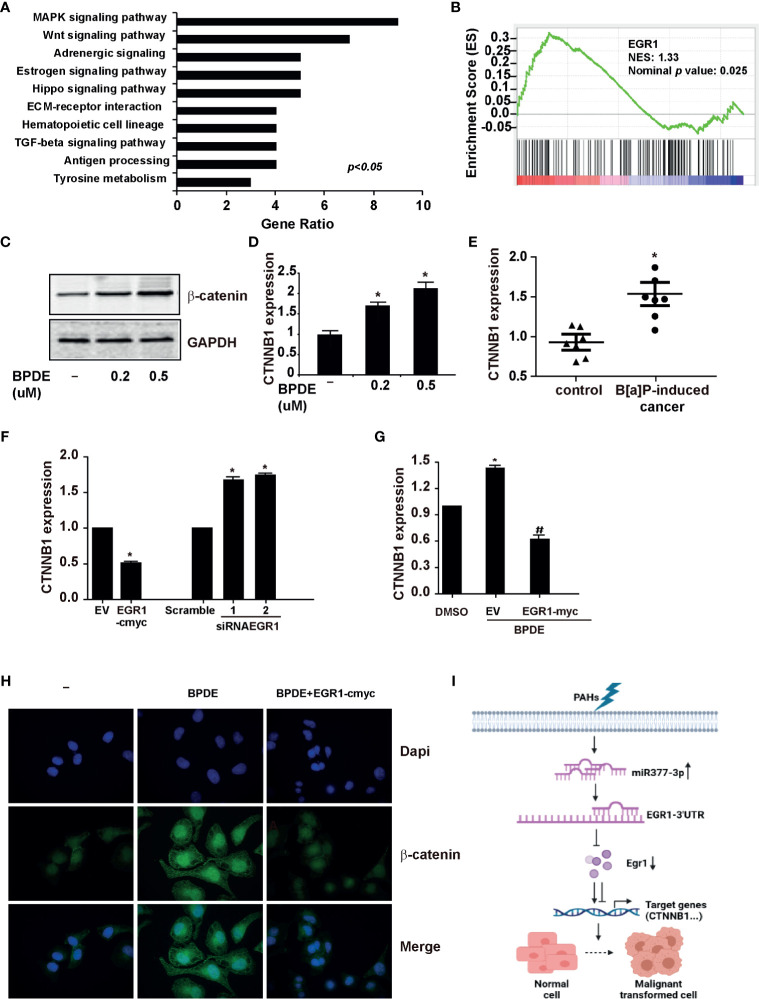
EGR1 inhibition was involved in the regulation of Wnt/β-catenin transduction in PAH-induced tumorigenesis. **(A, B)** KEGG analysis of differentially expressed genes and Gene set enrichment plots of differentially expressed genes belonging to the Wnt pathway in EGR1 knockdown cells. **(C, D)** β-catenin protein content and mRNA gene expression of CTNNB1 in BPDE-induced transformed cells. **(E)** mRNA level of CTNNB1 gene in mice primary lung cancer tissues. **(F, G)** CTNNB1 gene expression in BEAS-2B cells upon EGR1 overexpression and knockdown and in BPDE-induced transformed cells upon EGR1 overexpression. **(H)** Immunostaining of β-catenin in malignant transformed cells with or without EGR1 overexpression. **(I)** Schematic diagram of the regulatory mechanism of miRNA-377-3p/EGR1 axis in tumorigenesis. The analyses were repeated three times, and the results were expressed as mean ± SD. ^#^,*P < 0.05.

EGR1 is an important transcription factor for regulating the cell cycle, differentiation, apoptosis, and stress. To identify the potential EGR1-downstream genes involved in the BPDE/B[a]P-induced tumorigenesis, we performed the RNA-sequencing by knocking down EGR1 expression. As expected, Kyoto Encyclopedia of Genes and Genomes (KEGG) analysis indicated that the Wnt/β-catenin pathway is one of the most significantly altered gene set concepts in EGR1 knockdown cells, and gene set enrichment analysis (GSEA) revealed a large fraction of Wnt/β-catenin downstream genes that displayed significant alterations (**Figures 6A, B** **)**. Moreover, we also observed the upregulation of β-catenin in BPDE-induced malignant transformed cells and mice primary lung cancer tissue (**Figures 6C–E**). By transient transfection, EGR1 overexpressing led to a reduction of CTNNB1 gene expression. Moreover, EGR1 knockdown upregulated the CTNNB1 gene expression (**Figure 6F**). Also, the rescue of EGR1 expression abrogated the upregulation and nuclear localization of β-catenin induced by BPDE exposure (**Figures 6G, H** **)**. These data suggested that the Wnt/β-catenin pathway is the potential downstream signal in EGR1-mediated cell malignant transformation.

Furthermore, we detected the most altered genes of RNA-sequencing by knocking down EGR1 in the transformed cells. Our result revealed that ATF3 and ANKRD1 were downregulated in malignant cells and mice primary lung cancer tissues induced by PAHs (**Figures S5C, D**). Ectopic expression of EGR1 resulted in the upregulation of ATF3 and ANKRD1. Moreover, siRNA of EGR1 reduced the expression of ATF3 and ANKRD1 (**Figure S5E**). Besides, the rescue of EGR1 expression in BPDE-induced transformed cells abrogated the inhibition of ATF3 and ANKRD1 expression induced by BPDE (**Figure S5F**). To sum up, our data indicated that the downregulation of EGR1 could alter the downstream cell signals and the expression of its target genes to contribute to the cell malignant transformation and lung carcinogenesis.

## Discussion

B[a]P can directly induce lung carcinogenesis by inducing DNA damage and activating the signaling pathways (26–28). This study further investigated early events and the molecular mechanisms of gene dysregulation that lead to cell malignant transformation and lung tumorigenesis following B[a]P/BPDE exposure. We discovered that B[a]P/BPDE treatment led to miR-377-3p induction, which targeted EGR1-3’UTR and inhibited its expression, subsequently resulting in the activation of Wnt/β-catenin signal and promotion of cell malignant transformation, thus further contributing to lung tumorigenesis. Consequently, EGR1 could be considered a potential target for B[a]P initiation of lung carcinogenic actions.

As a transcription factor, EGR1 has a crucial role in human cancers. EGR1 has been increasingly attracting research attention due to its tumor-suppressing role in the occurrence and development of tumors. The expression of EGR1 decreases or even disappears in a variety of human malignancies, and its expression level is associated with tumor sensitivity to chemotherapy (29). EGR1 depletion has been associated with tumor anti-apoptotic and invasion events, whereas its overexpression may depress the tumorigenicity and metastasis in different cancer cells, including lung cancer (30). Mechanistically, EGR1 can directly transactivate P53 and PTEN, implicated in the proliferation inhibition of lung tumor cells (31, 32). It can also suppress the EMT transition and cell migration in lung cancer by regulating TGFβ activity (33). Recent studies have shown that EGR1 can directly and negatively regulate cell growth in different epithelial tumor cell lines (34). It can also regulate KRT18 expression to inhibit the malignancy of human NSCLC cells (35). Our data showed that EGR1 was strongly decreased in the early stage of malignant cell transformation upon BPDE exposure. The inhibition of EGR1 promoted the progression of BPDE-induced tumorigenicity. Moreover, the downregulation of EGR1 was also confirmed in B[a]P-induced lung tumors *in vivo*. The results indicated that EGR1 has a tumor repressive effect in cell malignant transformation and lung tumorigenesis upon B[a]P/BPDE treatment.

DNA methylation and miRNA dysregulation are important molecular mechanisms of gene expression, which are critical for epigenetic regulation in tumor formation and development by negatively regulating targeting downstream genes (36, 37). Recent studies reported that miR-301b, miR-191, and miR-146a could target EGR1 mRNA and inhibit its expression, thus contributing to oncogenesis (16, 38, 39). In this study, EGR1 was persistently decreased after BPDE exposure but without variation of its promoter DNA methylation level. miRNA screening analysis demonstrated that miR-377-3p is a new regulator of EGR1 by directly binding to its 3’UTR. miR-377-3p was significantly increased in BPDE-induced malignant transformed cells, as well as in the lung tumor tissues of B[a]P-treated A/J mice. Antagonized miR-377-3p reversed the effect of EGR1 in cell malignant transformation, thus supporting the critical role of miR-377-3p in regulating EGR1 expression to promote cell transformation and tumor formation. Recent studies reported that miR-377 displays an ambiguous role in different cancers. miR-377-3p can drive malignancy characteristics by upregulating GSK-3β expression and activating the NF-κB pathway in CRC cells (22). It can also target the pro-oncogenic genes, like E2F3, VEGF, and CDK6, or negatively regulate the Wnt/β-catenin signaling to suppress the proliferation of cancer cells (40–43). However, the dual effect of miR-377 in tumor inhibition and promotion needs to be further explored.

Clinical studies reported that the depletion of EGR1 sensitizes the chemotherapy of cisplatin in ovarian tumors (44). The low levels of EGR1, associated with the expression of PTEN, can predict poor outcomes after surgical resection of NSCLC (45). In this study, clinic tissue analysis showed a downregulation of EGR1 expression in cancer tissues compared with the normal tissue. The repression of EGR1 was associated with the local invasion depth, lymph nodes, and TNM stages. It was also negatively associated with histological grade (**Table 1**). Our result confirmed that the deactivation of EGR1 was associated with cancer aggressiveness.

Furthermore, it is reported that EGR1 can be increased by chemotherapy, and negatively regulate the Wnt/β−catenin signaling pathway in CML cells (46). In this study, we observed an enrichment of Wnt/β−catenin downstream genes after EGR1 knockdown. Besides, β−catenin, the key effector of canonical Wnt signaling, was activated in malignant transformed cells and lung cancer tissues following EGR1 inhibition. The rescue of EGR1 expression reversed the upregulation and nuclear staining of β−catenin after BPDE exposure. The Wnt/β−catenin pathway is a cell signaling that promotes cancer initiation and development. It has an important role in crucial cellular processes, including cell fate determination, embryonic development, homeostasis, motility, polarity, and stem cell renewal (47). It has also been reported that the activation of canonical Wnt/β-catenin signaling is critical for the initiation and progression of NSCLC (48). In patient-derived xenograft models of lung cancer, the activation of WNT/β-catenin signaling and nuclear β-catenin staining was associated with a poor prognosis in patients with lung cancer (49). Previous studies also reported that the Wnt/β-catenin pathway contributes to the induction of EMT by transactivating several EMT-related transcriptional factors, such as Snail, Slug, Twist, ZEB1, and ZEB2 in lung adenocarcinoma (50). Moreover, we also observed that ANKRD1 and ATF3, as the target genes of EGR1, were significantly downregulated in malignant transformed cells and mice lung cancer tissues. ATF3, a highly conserved transcription factor, was described as a principal target of EGR1 and discussed as a tumor suppressor and promoter (51–53). A recent study also reported that ATF3 and EGR1 are involved at the beginning of the inflammatory processes related to cancer (54). ANKRD1 is a tumor-suppressive downstream gene of the Hippo pathway, downregulated in different human cancers (55, 56). A previous study demonstrated that ANKRD1 could be inhibited by lncRNA, resulting in the promotion of pancreatic cancer proliferation and metastasis (57). Therefore, EGR1 could regulate its downstream signals and target genes, thus having a tumor-suppressive role in human lung cancer.

In summary, our current study demonstrated the regulation mechanism of EGR1 inhibition induced by miR-377-3p activation following exposure to environmental carcinogen B [a]P/BPDE. We also discovered that EGR1 has a repressive effect on lung tumorigenesis by regulating the Wnt/β-catenin signaling pathway (**Figure 6I**). Our findings provided a novel molecular regulatory mechanism through which the miR-377-3p/EGR1 axis was implicated in cell malignant transformation and tumorigenesis induced by PAH.

## Data Availability Statement

The datasets presented in this study can be found in online repositories. The names of the repository/repositories and accession number(s) can be found in the article/**Supplementary Material**.

## Ethics Statement

The studies involving human participants were reviewed and approved by the ethics committee of the Second Affiliated Hospital of Zhejiang University. The patients/participants provided their written informed consent to participate in this study. The animal study was reviewed and approved by The Committee on the Use of Animals of Zhejiang University.

## Author Contributions

XK: Conceptualization, Investigation, Resources, and Writing - original draft. LH and RW: Investigation, Methodology, and Validation. YS and LF: Software, Validation. ZW: Methodology, Validation. JShe: Review and Editing, Supervision, and Project administration. JSha: Review and Editing, Resources, and Supervision. HQ: Writing - Review and Editing, Investigation, Methodology, Formal analysis, Project administration, and Funding acquisition. All authors contributed to the article and approved the submitted version.

## Funding

This work was supported by the Zhejiang Provincial Natural Science Foundation of China (No. LY18H160024, LY20H160040), the National Natural Science Foundation of China (No. 81472543, No. 81772919), the National Key R&D Program of China (2016YFC1303401), and the Zhejiang Medical and Health Science and Technology Foundation (2018KY119).

## Conflict of Interest

The authors declare that the research was conducted in the absence of any commercial or financial relationships that could be construed as a potential conflict of interest.

## Publisher’s Note

All claims expressed in this article are solely those of the authors and do not necessarily represent those of their affiliated organizations, or those of the publisher, the editors and the reviewers. Any product that may be evaluated in this article, or claim that may be made by its manufacturer, is not guaranteed or endorsed by the publisher.
