# Supplementary material for: miR-377-3p-Mediated EGR1 Downregulation Promotes B[a]P-Induced Lung Tumorigenesis by Wnt/Beta-Catenin Transduction
Source: Front Oncol. 2021 Aug 23;11:699004. doi: 10.3389/fonc.2021.699004 (PMC8419355; doi:10.3389/fonc.2021.699004)
Supplement: Supplementary file 1 [file DataSheet_1.pdf]

Supplementary Figure legends

Figure S1. BPDE decreased EGR1 in malignant transformed cells. (A) Top, representative images of transwell assays; bottom, relative numbers of migration cells. (B) Top, representative images of wound healing assays; bottom, percentage wound closure of migration cells. (C) RNA-sequencing profiling of the 51 genes that are differentially expressed among DMSO-treated BEAS-2B cells and BPDE-treated BEAS-2B cells. Genes expression shown as pseudocolor scale (−2.0 to 2.0) with red or blue denotes the high or low expression levels, respectively. The genes selected with the FDR<0.01, Fold change>2, P value<0.05.

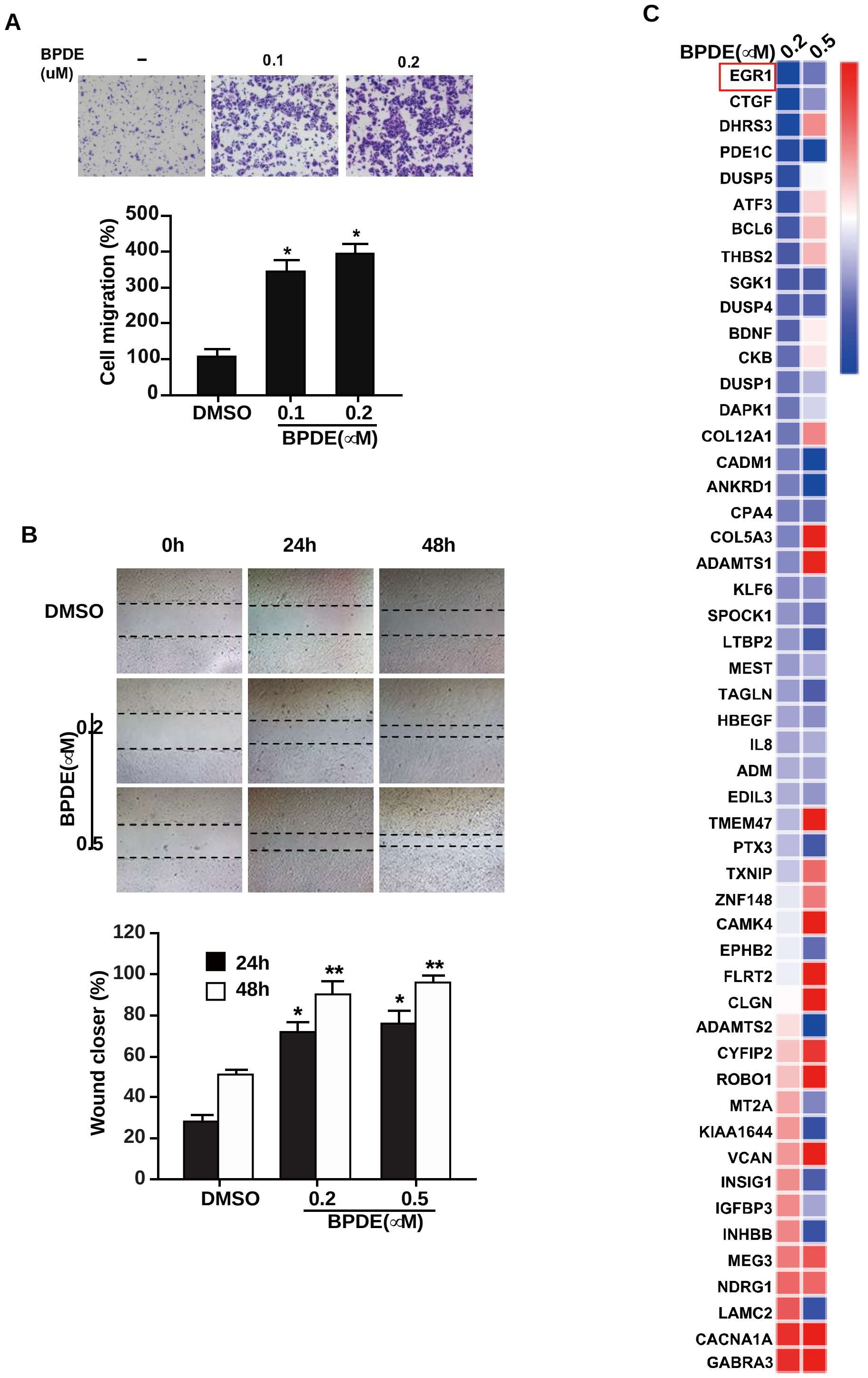
A
BPDE (uM)
–
0.1
0.2
Cell migration (%)
500
400
300
200
100
0
*
*
DMSO
0.1
0.2
BPDE(∝M)
B
0h
24h
48h
DMSO
BPDE(∝M)
0.2
0.5
Wound closer (%)
120
100
80
60
40
20
0
24h
48h
*
**
*
**
DMSO
0.2
0.5
BPDE(∝M)
C
BPDE(∝M) 0.2 0.5
EGR1
CTGF
DHRS3
PDE1C
DUSP5
ATF3
BCL6
THBS2
SGK1
DUSP4
BDNF
CKB
DUSP1
DAPK1
COL12A1
CADM1
ANKRD1
CPA4
COL5A3
ADAMTS1
KLF6
SPOCK1
LTBP2
MEST
TAGLN
HBEGF
IL8
ADM
EDIL3
TMEM47
PTX3
TXNIP
ZNF148
CAMK4
EPHB2
FLRT2
CLGN
ADAMTS2
CYFIP2
ROBO1
MT2A
KIAA1644
VCAN
INSIG1
IGFBP3
INHBB
MEG3
NDRG1
LAMC2
CACNA1A
GABRA3

Figure S1

Figure S2. B[a]P-induced lung tumorigenesis in A/J mice. (A) A model of B[a]P induced murine lung tumorigenesis. (B) PET/CT imaging of B[a]P-treated A/J mice. (C) Summary of the numbers of mice and the rate of tumor formation in B[a]P-treated A/J mice.

A

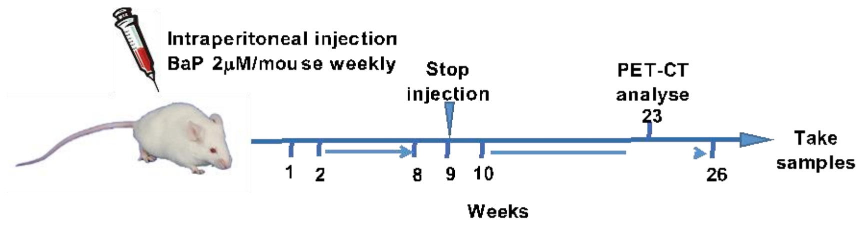

B

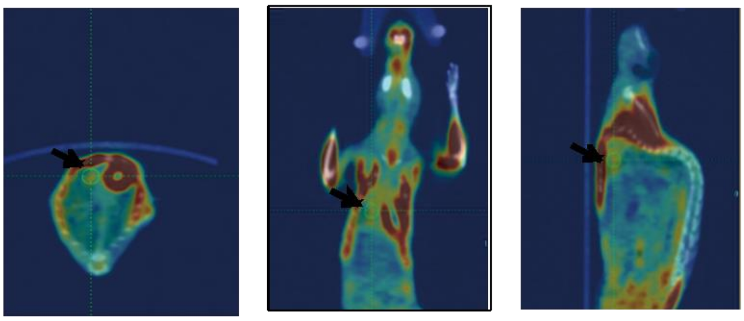

C

	A/J mouse N.	Death N.	Tumor incident N.	Tumor formation Rate(%)	Tumor N.
BaP treated	25	3	17	77.3	5±2.89
Contol	6	0	0	0	0
Total	31	3	17	77.3	5±2.89

Figure S2

Figure S3. EGR1 inhibition was observed in clinic lung cancer tissues. (A) EGR1 mRNA expression in 8 paired cases of human lung cancer tissues. A and S: patient's number. (B,C) Analysis of two datasets supported in Lung Cancer Explorer of EGR1 mRNA expression in lung cancer and normal tissues. (D) Methylation status at CpG sites in EGR1 promoter by DNA BSP analysis.

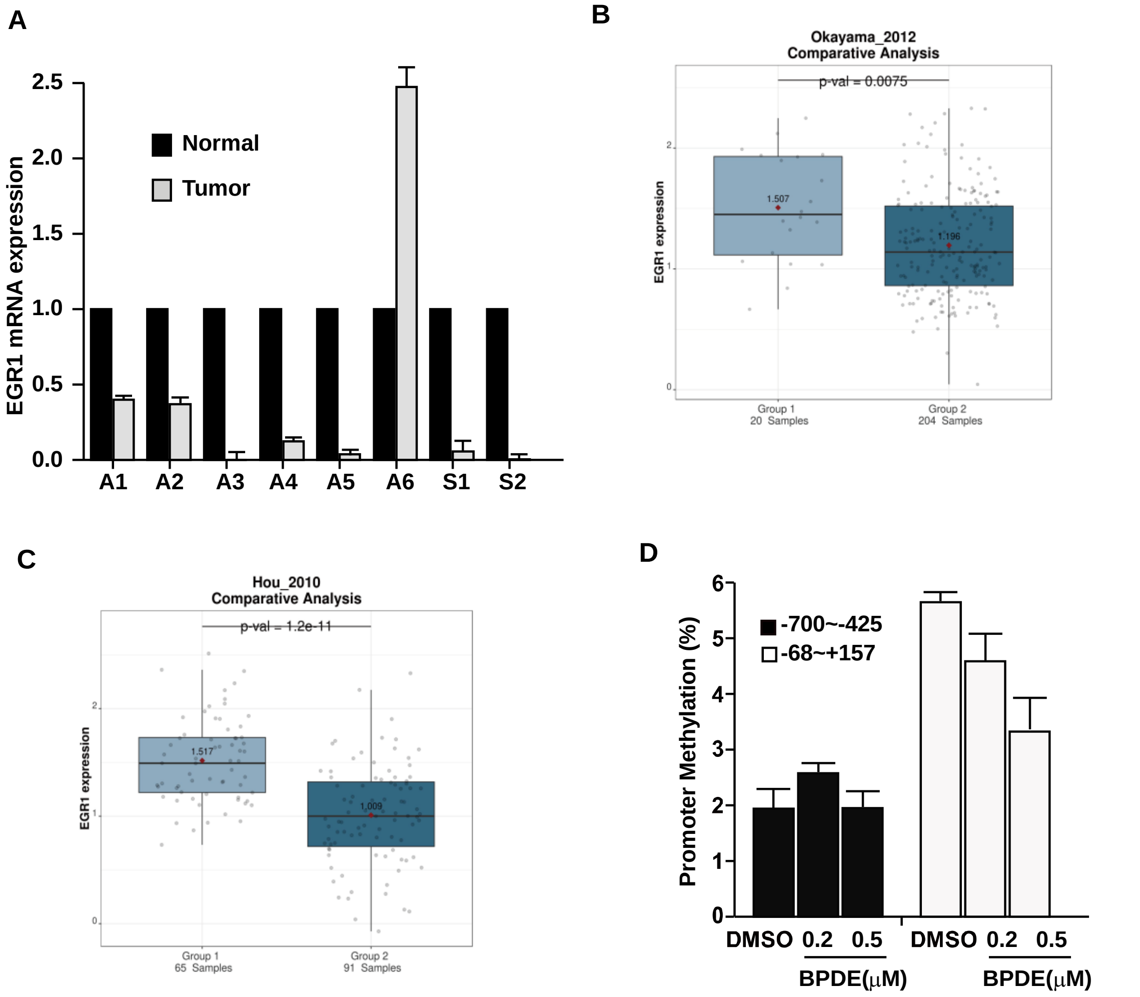

Figure S3

Figure S4. EGR1 implicated an important role in BPDE-induced cell malignant transformation. (A) Expression of EGR1 in DMSO or BPDE-treated BEAS-2B cells infected with EGR1 overexpressed lentivirus. (B) EGR1 expression in normal human lung epithelial BEAS-2B cells knockdown with sh-EGR1. (C) Soft-agar colony formation assays of BEAS-2B cells infected with scramble and sh-EGR1 lentivirus. Top, representative images of colony formation; bottom, quantitative results of cell colony per field. (D) Transwell migration assays. Top, representative images; bottom, quantitative results of migratory cells per field. (E) Wound healing assays. Left, representative images at 0, 24 h and 48 h after wounding; right, quantitative results of wound closure. (F,G) Rescue of EGR1 expression suppressed shEGR1-induced malignant transformation of normal human lung epithelial BEAS-2B cells. Soft-agar colony formation assays. Left, representative images; right, quantitative results of cell colony per field. Transwell migration assays. Left, representative images; right, quantitative results of migratory cells per field.

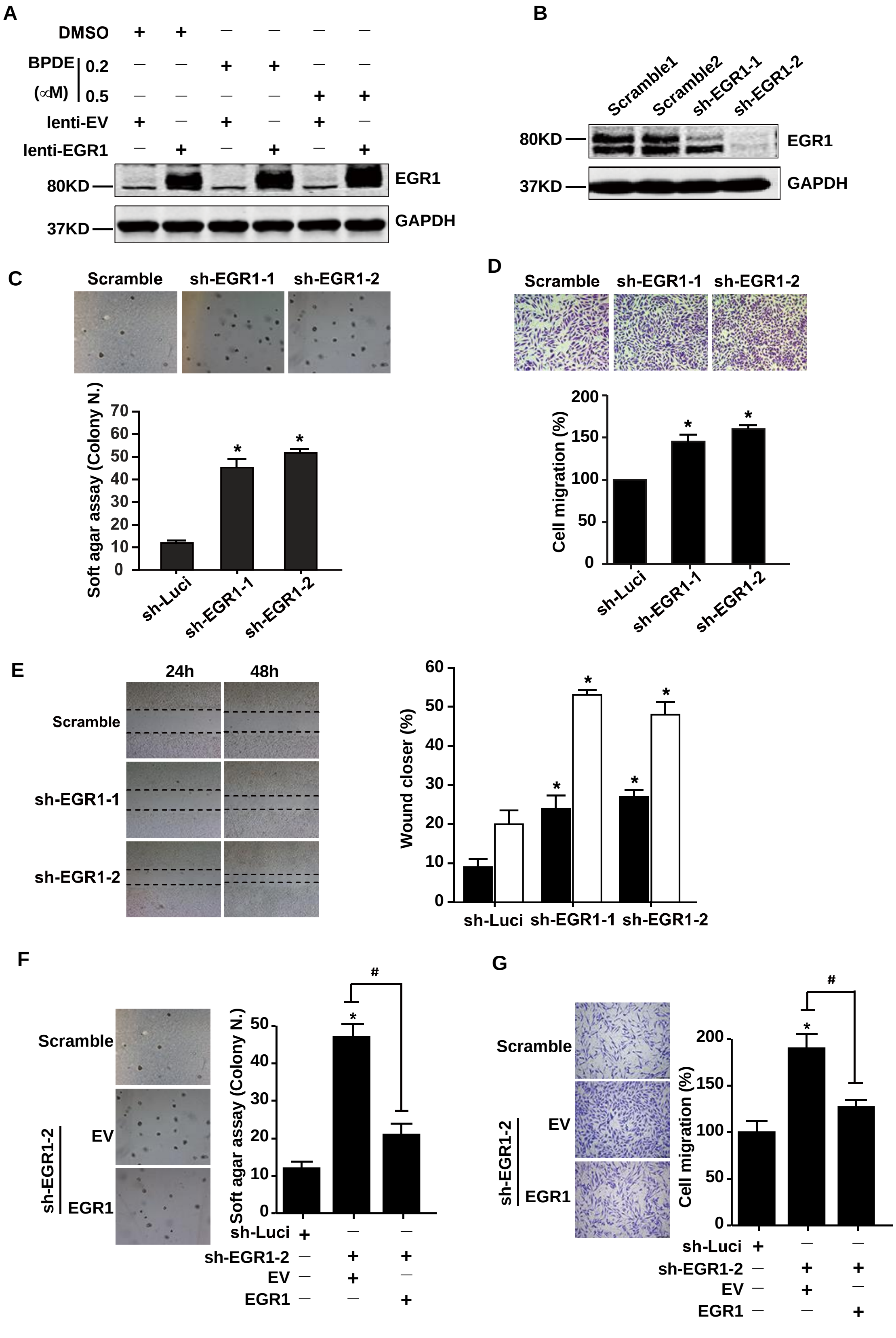
A
DMSO
BPDE
(∝M)
0.2
0.5
lenti-EV
lenti-EGR1
80KD
37KD
EGR1
GAPDH
B
Scramble1
Scramble2
sh-EGR1-1
sh-EGR1-2
80KD
37KD
EGR1
GAPDH
C
Scramble
sh-EGR1-1
sh-EGR1-2
Soft agar assay (Colony N.)
sh-Luci
sh-EGR1-1
sh-EGR1-2
D
Scramble
sh-EGR1-1
sh-EGR1-2
Cell migration (%)
sh-Luci
sh-EGR1-1
sh-EGR1-2
E
24h
48h
Scramble
sh-EGR1-1
sh-EGR1-2
Wound closer (%)
sh-Luci
sh-EGR1-1
sh-EGR1-2
F
Scramble
sh-EGR1-2
EV
EGR1
Soft agar assay (Colony N.)
sh-Luci
sh-EGR1-2
EV
EGR1
G
Scramble
sh-EGR1-2
EV
EGR1
Cell migration (%)
sh-Luci
sh-EGR1-2
EV
EGR1

Figure S4

Figure S5. EGR1 was decreased in clinic lung cancer tissues and it can regulate the downstream target genes expression. (A) The correlation analysis of EGR1 and miR-377-3p mRNA expression in fresh clinic lung cancer tissues. (B) Representative image of IHC staining of EGR1 in human paired lung cancer and adjacent normal tissues. (C,D) mRNA expression of ATF3, ANKRD1 in BPDE-induced malignant transformed cells and B[a]P-treated murine lung tumors tissues. (E) Overexpression of EGR1 or knockdown of EGR1, ATF3, ANKRD1 mRNA expression in BEAS-2B cells. (F) ATF3, ANKRD1 mRNA expression in BPDE-induced transformed cells underlying EGR1 overexpression.

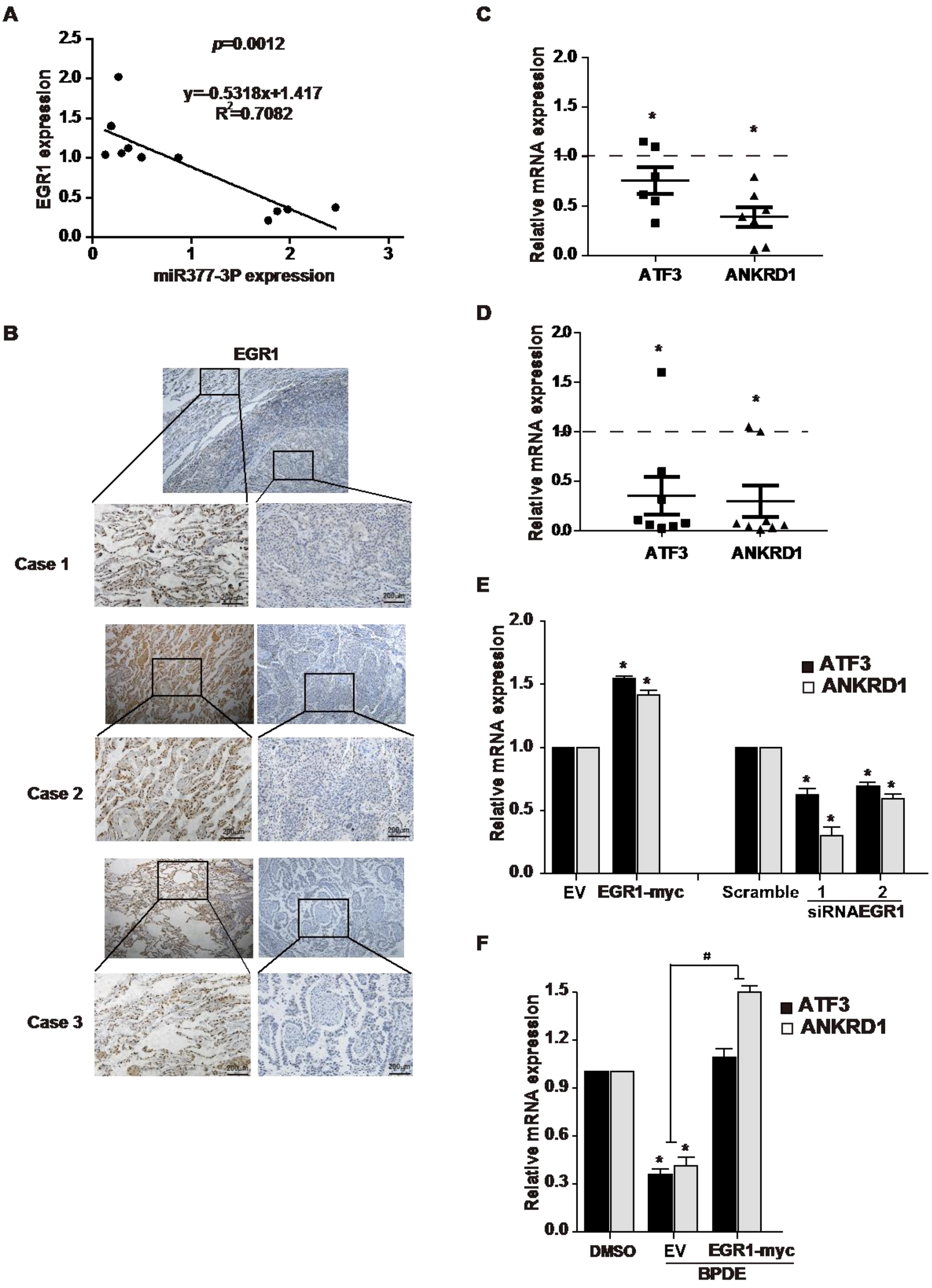
A
p=0.0012
y=-0.5318x+1.417
R2=0.7082
EGR1 expression
miR377-3P expression
B
EGR1
Case 1
Case 2
Case 3
C
Relative mRNA expression
ATF3
ANKRD1
D
Relative mRNA expression
ATF3
ANKRD1
E
Relative mRNA expression
ATF3
ANKRD1
EV
EGR1-myc
Scramble
1
2
siRNAEGR1
F
Relative mRNA expression
ATF3
ANKRD1
DMSO
EV
EGR1-myc
BPDE

Figure S5
