# Supplementary material for: miR-377-3p-Mediated EGR1 Downregulation Promotes B[a]P-Induced Lung Tumorigenesis by Wnt/Beta-Catenin Transduction
Source: Front Oncol. 2021 Aug 23;11:699004. doi: 10.3389/fonc.2021.699004 (PMC8419355; doi:10.3389/fonc.2021.699004)
Supplement: Supplementary file 2 [file DataSheet_2.pdf]

Supplementary Methods

Cell proliferation analysis

Cells ($1.2x10^4$ cells/well) were seeded in 24-well plates and cultured during either: 24, 48, 72, 96 and 120 hours. Cells were digested by trypsin and re-suspended in fresh medium and the cell number was counted every 24 hours.

RNA-Seq Analysis

Total RNA was isolated from cells treated BPDE or EGR1 knock down and control cells using TRIzol reagent (Life Technologies). The purity of total RNA was assessed with a spectrophotometer (NanoDrop ND-1000), and the integrity of total RNA was judged through 1% formaldehyde denatured gel electrophoresis. Then, the RNA of each sample was used for RNA-Seq and performed by Novogene Corporation. For gene expression analysis, HT Seq v0.6.1 was used to count the read numbers mapped of each gene. And then, RPKM (Reads Per Kilo bases per Million reads) was calculated based on the length of the gene and reads count mapped to this gene. Corrected q-value of 0.05 and log2 (Fold change) of 1 were set as the threshold for significant differential expression.

Cell Culture and treatment

Human bronchial epithelial NL20 cell line was purchased from American Type Culture Collection (ATCC) in March 2016 and cytogenetically tested and authenticated before the cells were frozen. Human lung cancer cells A549, H1299, H446, H520, HBE were purchased from the Cell Bank of the Chinese

Academy of Science (Shanghai, China) in December 2015. The authenticity of cell lines in our study had been verified by DNA sequencing using the Applied Biosystems AmpF/STR Identifier kit. All cell lines had never been passaged longer than 4 months and last checked in September 2017. Cells were cultured in RPMI 1640 (Gibco) supplemented with 10% Fetal Bovine Serum, (Gibco),streptomycin (100μgm/L) and penicillin (100U/mL1) at 37 ℃ and 5% CO2. For exposure, the cells were seeded in triplicate 24 hours prior to exposure. The final concentrations of urban dust suspension were 50 and 100, respectively. QRT-PCR analysis was performed 6, 12 and 24 hours after the exposure.

DNA Bisulfite sequencing PCR

Total DNA was isolated by Qiagen DNA isolation kit from cells either BPDE treated or not. And then, DNA BSP analysis was performed by Xiangyin Biological Corporation.
